# Fiber-Based Electrochemical Biosensors for Monitoring pH and Transient Neurometabolic Lactate

**DOI:** 10.1021/acs.analchem.0c05108

**Published:** 2021-04-02

**Authors:** Marsilea A. Booth, Sally A. N. Gowers, Melinda Hersey, Isabelle C. Samper, Seongjun Park, Polina Anikeeva, Parastoo Hashemi, Molly M. Stevens, Martyn G. Boutelle

**Affiliations:** † Department of Bioengineering, Imperial College London, London SW7 2AZ, U.K.; ‡ Department of Materials, Imperial College London, London SW7 2AZ, U.K.; § Institute of Biomedical Engineering, Imperial College London, London SW7 2AZ, U.K.; ∥ Department of Chemistry, University of South Carolina, Columbia, South Carolina 29208, United States; ⊥ Department of Electrical Engineering and Computer Science, Massachusetts Institute of Technology, Cambridge, Massachusetts 02139, United States; # Department of Bio and Brain Engineering, Korea Advanced Institute of Science and Technology (KAIST), Daejeon 34141, Republic of Korea; ¶ KAIST Institute for Health Science and Technology, Daejeon 34141, Republic of Korea; ∇ Department of Materials Science and Engineering, Massachusetts Institute of Technology, Cambridge, Massachusetts 02139, United States

## Abstract

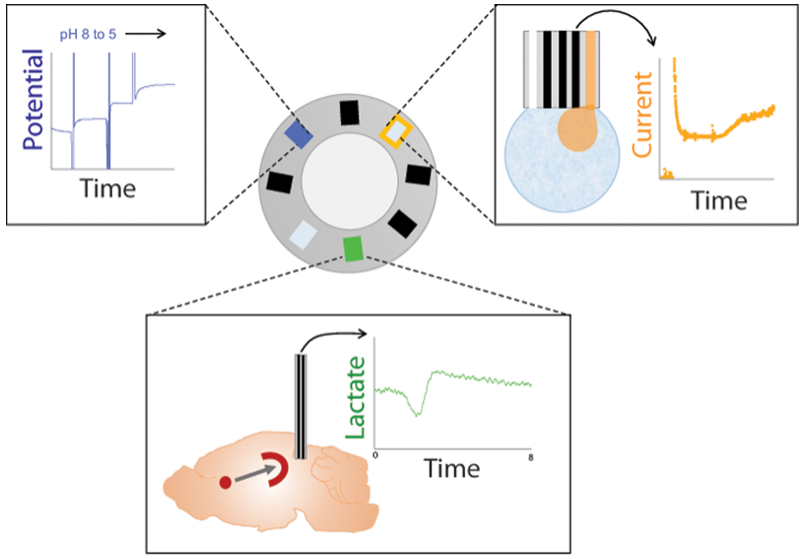

Developing tools that are able to monitor transient neurochemical dynamics is important to decipher brain chemistry and function. Multifunctional polymer-based fibers have been recently applied to monitor and modulate neural activity. Here, we explore the potential of polymer fibers comprising six graphite-doped electrodes and two microfluidic channels within a flexible polycarbonate body as a platform for sensing pH and neurometabolic lactate. Electrodes were made into potentiometric sensors (responsive to pH) or amperometric sensors (lactate biosensors). The growth of an iridium oxide layer made the fiber electrodes responsive to pH in a physiologically relevant range. Lactate biosensors were fabricated via platinum black growth on the fiber electrode, followed by an enzyme layer, making them responsive to lactate concentration. Lactate fiber biosensors detected transient neurometabolic lactate changes in an in vivo mouse model. Lactate concentration changes were associated with spreading depolarizations, known to be detrimental to the injured brain. Induced waves were identified by a signature lactate concentration change profile and measured as having a speed of ∼2.7 mm/min (*n* = 4 waves). Our work highlights the potential applications of fiber-based biosensors for direct monitoring of brain metabolites in the context of injury.

## Introduction

Chemical monitoring of human tissue for health is becoming an increasingly critical target.^1,2^ Focusing particularly on the brain, being able to monitor dynamic changes in neurochemicals is an important pursuit.^3^ Microdialysis is an FDA-approved sampling technique to monitor human tissue in the clinic.^4^ It is able to measure multiple analytes at a single probe since the sensors are located outside the body. This external location also means that sensor calibration is more straightforward than calibration of implanted sensors. Microdialysis has been coupled to online analysis systems to provide continuous measurements in real time.^5,6^ Due to the requirement of a certain length of tubing to separate the probe from the sensors, this type of microdialysis is accompanied by a time delay and, therefore, diffusion-based broadening of transient concentration changes. The relatively large diameter of clinical microdialysis probes can damage tissue, invoking a foreign body response after implantation,^7^ an effect that can be mitigated by retrodialysis of anti-inflammatory agents such as dexamethasone,^8^ or using custom microfabricated probes with reduced dimensions.^9,10^

An alternative approach for making chemical measurements in tissue is the use of implantable electrochemical sensors. Most implantable sensors offer improved temporal resolution^11^ and a smaller footprint than microdialysis.^12,13^ However, implanted sensors have several drawbacks. First, these sensors typically offer single analyte measurement, although manifold sensors or sensor arrays are able to perform multianalyte measurements.^14^ Second, calibration of implanted sensors is an ongoing challenge in the community, an issue that complicates accurate interpretation of in vivo measurements. Third, implantable sensors lack the capability to recover sample aliquots and to deliver drugs locally. Finally, Young’s modulus (ability of a material to withstand changes in length under compression) for these sensors is significantly different from that of brain tissue.

Hybrid multifunctional probes are an emerging strategy that can combine the best aspects of both microdialysis and implanted sensors, thereby overcoming technological limitations. Microfabrication has been used to create multifunctional and multiplexed devices.^15−17^ One study combined microdialysis with optogenetics to monitor extracellular concentrations of glutamate and dopamine in the medial prefrontal cortex of rodents after stimulation.^15^ In other work, Altuna et al. developed a multielectrode device with capacity for neural recording and drug delivery via a microfluidic channel.^16^ A microelectrode array coupled to a polydimethylsiloxane microfluidic channel by Wang et al. demonstrated multianalyte sensing and local chemical delivery.^17^ Multifunctional probes provide the capability for multianalyte measurements in a single device and the potential for in situ calibration; however, many examples of these devices require labor-intensive fabrication and do not overcome Young’s modulus issue because they are rigid.

Thermal drawing is an elegant fabrication process that incorporates multiple functionalities in a single entity. The process is initiated by the construction of a macroscale model preform (template) containing all the elements required in the final fiber. Thermally drawing this preform allows retention of components, together with miniaturization and scalable production.^18,19^ Similar fibers have been used in prior work for detecting neuronal signals, cell growth, and optogenetic neuromodulation.^19−21^ Using this approach, polymer materials can be used to impart flexibility to the probe. Compared to similarly sized insulated steel microwires, polymer fibers have been found to evoke lower foreign body response after implantation,^19^ implying a good level of in vivo biocompatibility. Moreover, the fabrication of multifunctional fibers using a thermal drawing process brings forth the ability to perform multiple electrochemical measurements and fluidics in a single device.

This work describes the design, construction, and development of fiber-based biosensors with multiple capabilities. Here, we utilize thermally drawn fibers containing six electrodes fabricated from the combination of conductive polyethylene and graphite powder and two microfluidic channels within a polycarbonate body.^19^ Although these fibers have been previously applied to monitor and modulate neural activity in the brain of moving mice,^19^ their potential as biochemical sensors remains to be explored. We demonstrate the microelectrode behavior of the fiber electrodes and show the multifunctional nature of the fibers by flowing different solutions through the internal microfluidic channels and monitoring the response at the electrode surface. We validate the functionality of a potentiometric pH sensor and an amperometric lactate biosensor. We have previously shown that sensing neurochemical and electrophysiological signals in the brain and peripheral tissue gives valuable insights into brain function and disease pathophysiology.^5,6,19,21−25^ Monitoring these different analytes requires different approaches (potentiometric sensors for pH and potassium and amperometric sensors for glucose and lactate). This work is a proof-of-concept study illustrating the validity of using a flexible fiber as either potentiometric or amperometric electrochemical sensors. Furthermore, the ability of the lactate sensors is demonstrated in mice to measure physiologically relevant changes in vivo. New sensing tools, such as described here, capable of real-time measurement of neurochemical dynamics in the brain have great potential in the pursuit to measure brain function and for diagnostics.

## Experimental Section

### Materials and Reagents

Lactate oxidase (20–60 U/mg) from *Aerococcus viridans* was obtained from Sekisui Enzymes. All other reagents were obtained from Sigma-Aldrich. Phosphate-buffered saline (PBS) was a physiological saline chosen to match the artificial cerebral spinal fluid of the brain buffered to pH 7.4.

### Design and Fabrication of the Fiber

Design and fabrication of the fiber has been detailed elsewhere.^19^ In brief, a macroscopic template preform was made comprising six electrodes formed from a custom conductive polymer composite (conductive polyethylene and 5% graphite), two microfluidic channels, and an optical waveguide. The optical waveguide was not used in this work. A thermal drawing process heated the preform near glass transition temperature and stretched it into ∼100 m-long fiber, reducing feature dimensions by 50–200-fold.^19^ The final fiber is flexible and can be electrically connectorized for individual electrodes and fluidic channels. This was performed in a similar manner to that described elsewhere;^19^ however, fluidic connection was also made from the end of the fiber as the optical component was not necessary in these experiments (Figure S1). The connectorization process is highly challenging due to the miniature and multiple-component nature of the fiber. However, once a fiber is connectorized, the end can be sliced with a sharp razor blade to cleanly expose a fresh surface, enabling a reusable fiber.

### Microfluidic Flow Experiments

The two microfluidic channels were individually addressed. Solution was pushed through each of the microfluidic channels at a speed of 1 μL/min under the control of a microfluidic pump (Hamilton). Through the first channel flowed PBS, while the second channel had ferrocene monocarboxylate solution, named Fc solution (1.5 mM in 100 mM sodium citrate, 150 mM sodium chloride and 1 mM EDTA). The flow was manually controlled between the two channels. Due to the miniature size of the channels (ca. 15 μm^19^), blocking occasionally occurred and there was difficulty in connectorizing the microfluidic channels.

For the flow experiments, a reference and counter electrode (Ag|AgCl and stainless steel, respectively) were placed nearby the fiber end, and the electrodes were held at +0.5 V versus Ag|AgCl. When the solution flowed through the channels, a droplet of the liquid formed, eventually bridging the electrodes enabling electrical contact. A video was taken continuously as this happened.

### pH Sensor Fabrication

An iridium oxide film was deposited on a carbon electrode to fabricate a pH sensor. A solution, as described by Yamanaka,^26^ of iridium tetrachloride hydrate (4.5 mM), hydrogen peroxide (30% w/w), and oxalic acid dihydrate (0.5 g) in water was used. Anhydrous potassium carbonate was added to adjust pH to 10.5. The solution was then left standing at room temperature for 60 h until a deep blue-violet coloration appeared. When not in use, the solution was stored in the fridge.^27^ Using this solution, a film was grown by applying an amperometric wave form of 300 s at 0.95 V, 10 min at open circuit, 300 s at 0.95 V, 10 min at open circuit, and finally 300 s at 0.95 V. Following rinsing, the pH sensor was used by measuring open circuit potential versus time under different pH conditions (10 mM PBS, pH adjusted by the addition of hydrochloric acid or sodium hydroxide as determined using a Mettler Toledo SevenEasy pH meter and Hanna Instruments pH probe).

### Electrochemical Deposition of Platinum Black

Platinizing solution consisted of 3 wt % chloroplatinic acid and 0.005 wt % lead acetate in deionized water. Amperometric deposition with a constant current density of −30 mA/cm^2^ was performed for 60 s (vs Ag|AgCl reference electrode with a large surface area platinum counter electrode) in a stirred solution. After rinsing, the electrode was cycled in H_2_SO_4_ (0.5 M) for 10 cycles between −0.2 and 1.3 V (scan rate 0.1 V/s). The electrode was then ready for further functionalization.

### Lactate Biosensor Fabrication

Lactate sensor fabrication was based on methods we have used for platinum microelectrodes detailed elsewhere.^6,28^ Namely, an exclusion layer of poly-*m*-phenylene diamine (100 mM in PBS, pH 7.4) is electrochemically deposited on to the electrode surface, followed by dip-coating of a hydrogel layer [of poly(ethylene glycol) diglycidyl ether] loaded with the lactate oxidase enzyme. After a curing period, the biosensor is ready for use or can be stored in the freezer. The lactate presence is detected using amperometry, where the working electrode is held at +0.7 V versus Ag|AgCl causing hydrogen peroxide oxidation. Sensor calibration in a beaker containing PBS (0.01 M PBS) solution allowed conversion of measured current to lactate concentration. Fitting was performed with the Michaelis–Menten equation, eq S1.

### Monitoring Lactate In Vivo

All animal procedures were carried out in accordance with the American laws for animal protection and institutional guidelines approved by the Institutional Animal Care and Use Committee (IACUC) at the University of South Carolina. Two male mice were anesthetized with an i.p. injection of 25% w/v urethane [Sigma-Aldrich Co., dissolved in 0.9% NaCl solution (Hospira)] during all surgical procedures. Temperature was maintained by heating pads below the mouse, routinely replaced as required throughout the experiment. Stereotaxic surgery was performed in order to drill small burr-holes in the frontal and parietal cortex for subsequent needle pricks. Two small holes were drilled at 3 mm posterior and 3 mm lateral, and 3 mm anterior and 3 mm lateral to Bregma on each hemisphere for needle pricks. Additionally, two holes were drilled 3 mm lateral to Bregma in each hemisphere for the lactate biosensor fibers. One biosensor (fiber) was placed in each hemisphere, with one electrode measuring lactate signal per fiber. Although holes were drilled and sensors were placed via stereotaxic positioning, needle pricks were performed by hand. Spreading depolarizations (SDs) are known to be induced by manual needle pricks^22,29^ and are not position-specific; therefore, stereotaxic equipment was not required. A chloridized Ag electrode was used as an Ag|AgCl reference electrode, while a stainless-steel counter electrode was used. These were both placed in the brain outside of the experimental region. The fiber was slowly lowered into the hole and connected. Biosensors were calibrated before and after implantation. Two in vivo experiments were performed. For experiment number 1, the right hemisphere contained an area of previously made local damage; meanwhile, the left hemisphere was untouched and remained fully intact. The local damage was caused by implantation and removal of a stimulation electrode for a separate experiment. For experiment number 2, a fiber with a lactate biosensor was placed in one hemisphere, while a fiber containing a control electrode (no lactate oxidase present^30^) was placed in the contralateral hemisphere. For both experiments, only one electrode was connected from each fiber biosensor due to the two-electrode measurement capacity of the two-channel wireless potentiostat used.

A waiting period of 60 min followed implantations in order to allow for stabilization. A needle prick was then performed. This needle prick gives a mechanically induced focal traumatic injury via a needle prick directly to the brain tissue.^8,22,29^ This in turn can give rise to SD waves. After a further stabilization period of 30 min, a needle prick in the other hemisphere was performed, and this continued as the animal conditions allowed, for eight needle pricks in total (experiment 1). Each hemisphere had a 60 min wait period between needle pricks. In the first animal, no SD wave was observed following the second needle prick. Therefore, a repeat needle prick after 10 min was performed in case an error had occurred. The expected SD wave followed. Control measurements including applying “non contact” needle pricks, jostling the reference/counter electrode, and aggressively touching the stereotaxic equipment were performed at the end of the experiment, and all showed no significant response (Figure S7). At the end of the experiment, the animals were humanely euthanized.

### Electrochemistry Instrumentation and Data Analysis

Three electrochemical instrumental setups were used. To characterize and perform lactate sensing and pH experiments, a 16-channel Powerlab analogue-to-digital converter (ADInstruments, Sydney, Australia) was coupled to in-house potentiostats, and data were recorded with LabChart software version 7.2 (ADInstruments, Sydney, Australia). For amperometric deposition of platinum black, a CompactStat (Ivium Technologies, the Netherlands) was used. For in vivo experiments, the setup has been detailed elsewhere.^31^ Briefly, an in-house built potentiostat was used for measurement, while wirelessly coupled via bluetooth to a tablet for data recording using in-house built software. Data analysis was performed using Lab chart (ADInstruments, Sydney, Australia) and smoothed with a Savitzky–Golay 101 or 201-point filter.

## Results and Discussion

### Fiber Fabrication and Electrode Characterization

**Figure 1:**
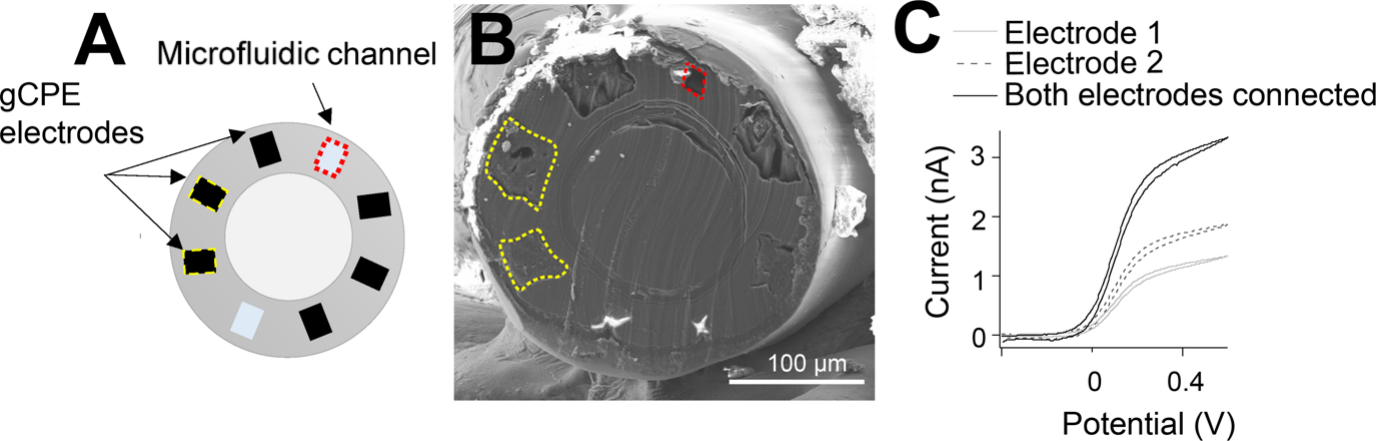
(A) Schematic showing the components of the fiber. Two of the six gCPE electrodes and one of the two microfluidic channels are highlighted in yellow and red, respectively. (B) SEM image of a razor blade-sliced fiber surface showing two electrodes highlighted in yellow and one of the microfluidic channels in red. One electrode and one microfluidic channel were damaged during the preparation process (as seen by charging); however, the remaining elements are functional. This image shows an example of the as-used fiber surface. (C) CVs of two bare electrodes in ferrocene monocarboxylate solution (1.5 mM, 10 mV s^–1^ vs Ag|AgCl) individually (grey and dashed lines) and when connected together (black line), indicating that they are individually addressable.

The drawn polycarbonate fiber has six electrodes and two microfluidic channels around the fiber edge,^19^Figure 1A,B. To expose a fresh surface, the fiber can be cut using a razor blade and the striations from this process can be seen in scanning electron microscope (SEM) images, a representative image shown in Figure 1B. Classic microelectrode behavior is found for a reversible solution redox species, Figure 1C. The electrodes are individually addressable as evidenced by an increase in current when two electrodes are connected; however, as can be seen, there is variability in the magnitude of individual electrode responses. A maximum of five of the six possible electrodes were connected within a single fiber for this work (Figure S2A) due to the challenge of miniature connectorization and not requiring all six electrodes; however, full connectorization is possible.^19^ Previous estimations published by Park et al.^19^ and SEM images suggest electrode dimensions of ∼400–1600 μm^2^. Estimates of active electrochemical areas based on the plateau current from Figure 1C suggest electrode dimensions of 42 and 85 μm^2^ for electrodes 1 and 2, respectively (eq S2). The fiber draw process, subsequent etching of a sacrificial outer layer, and fresh surface exposure can cause some geometric distortion of the electrodes and fluidic channels. This, together with the inhomogeneous nature of the 5% graphite and conductive polyethylene (gCPE) composite comprising the electrodes, explains why individual electrodes possess different electroactive surface areas, as seen in Figure 1C. Some variability is seen within fibers and across fibers, Figure S2B,C.

### Microfluidics and Electrochemical Detection

**Figure 2:**
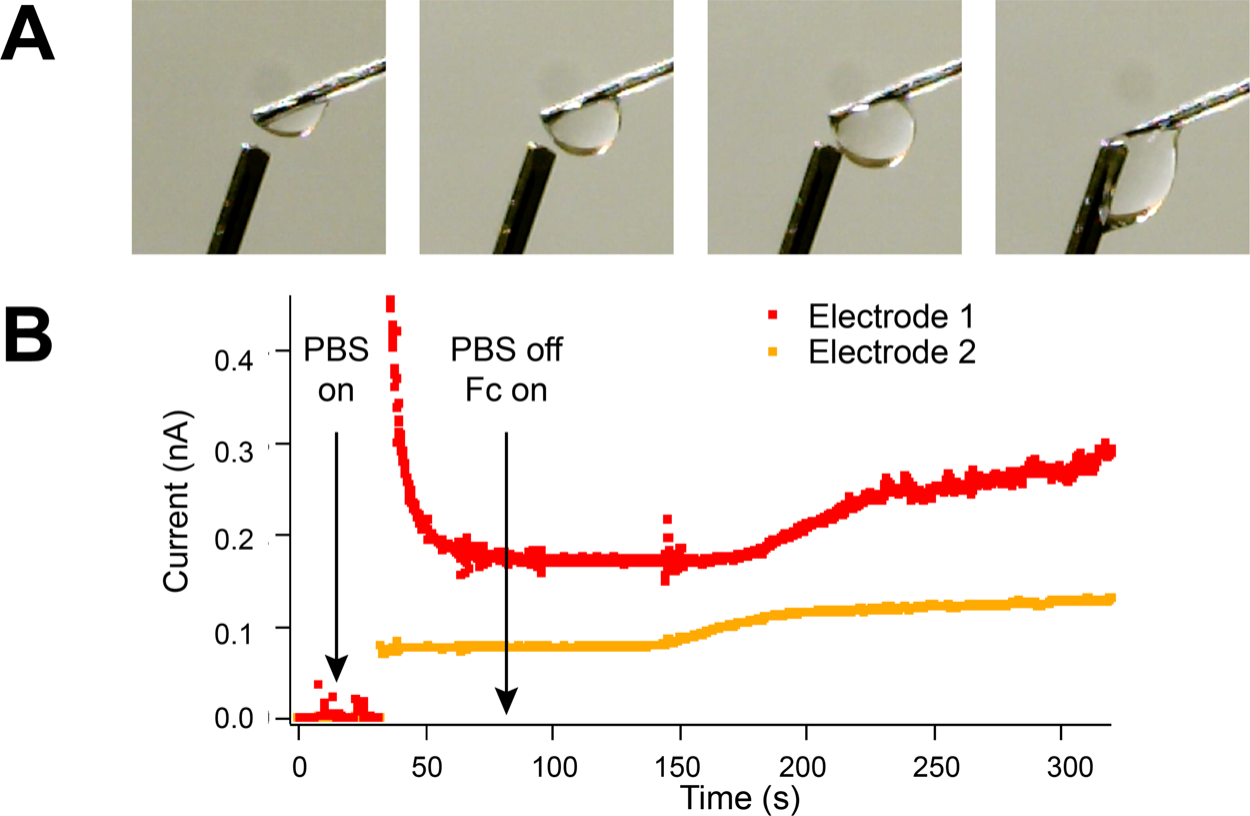
Fluidic experiment where solution is flowed through the microfluidic channels as shown by (A) images and (B) an overlay of current time traces measured at two electrodes in the single fiber containing the microfluidic channels. Images show the fiber working electrodes above and the reference/counter electrodes below (dark needle), and each image is sequentially 50 s apart. Initially, PBS flows through channel 1, and the air between the fiber electrode and reference/counter probe means no electrical connection. Once the liquid bridges the gap, connection is made and current flows. Channel 1 is then stopped, and channel 2 flows Fc solution, and a corresponding increase in current is observed as ferrocene is oxidized on the electrode surfaces. The delay in current increase arises due to the tubing connecting the fiber and pump and the fiber length. Differences in the observed current magnitude and charging currents between electrodes can be explained by fiber variation due to the inhomogeneous electrode composition, draw process, and preparation processes.

The multifunctional capability of the fiber can be shown using the fluidic channels and electrodes. Two solutions were flowed through the microfluidic channels independently. Electroactive solution (Fc solution) was flowed through one, while PBS through the other. A fiber with two active electrodes was placed nearby to a probe containing reference and counter electrodes (Ag|AgCl and stainless steel, respectively). When a sufficient droplet of the fluid had bridged the gap between the electrode and the probe, an electrical connection was made and current flowed, Figure 2. When the solution flow was changed from the PBS fluidic channel to the ferrocene-containing channel, an increase in current was observed as expected at two fiber electrodes, demonstrating the use of the microfluidics to characterize the electrochemical response. Variation in response between electrodes 1 and 2 can be seen in both magnitude and response time. The former can be explained by the inhomogeneous composition of the electrode material (5% graphite in gCPE), resulting in differing electroactive surface areas, as seen in Figures 1C and S2A. Meanwhile, variation in response time may be due to the position of the electrode relative to the fluidic channel and formed droplet. As can be seen in Figure 2A, the liquid droplet forms asymmetrically due to gravity and the angle of the fiber. Additionally, the fluidic channel is geometrically closer to some electrodes than others. Therefore, one electrode may be exposed to a higher concentration of ferrocene before the other, resulting in a difference in response time. The main contributor to changes in current are from faradaic processes, as seen by Figure S3, where a stable baseline of current is seen over 300 s when exposed only to PBS solution. For practical robust use of these fibers, the microfluidic channels require increased size through fiber optimization and improved connections to prevent blockages. However, the data reported here show the potential of such a system with fluidics and electrodes.

### Fibers for pH Sensing

**Figure 3:**
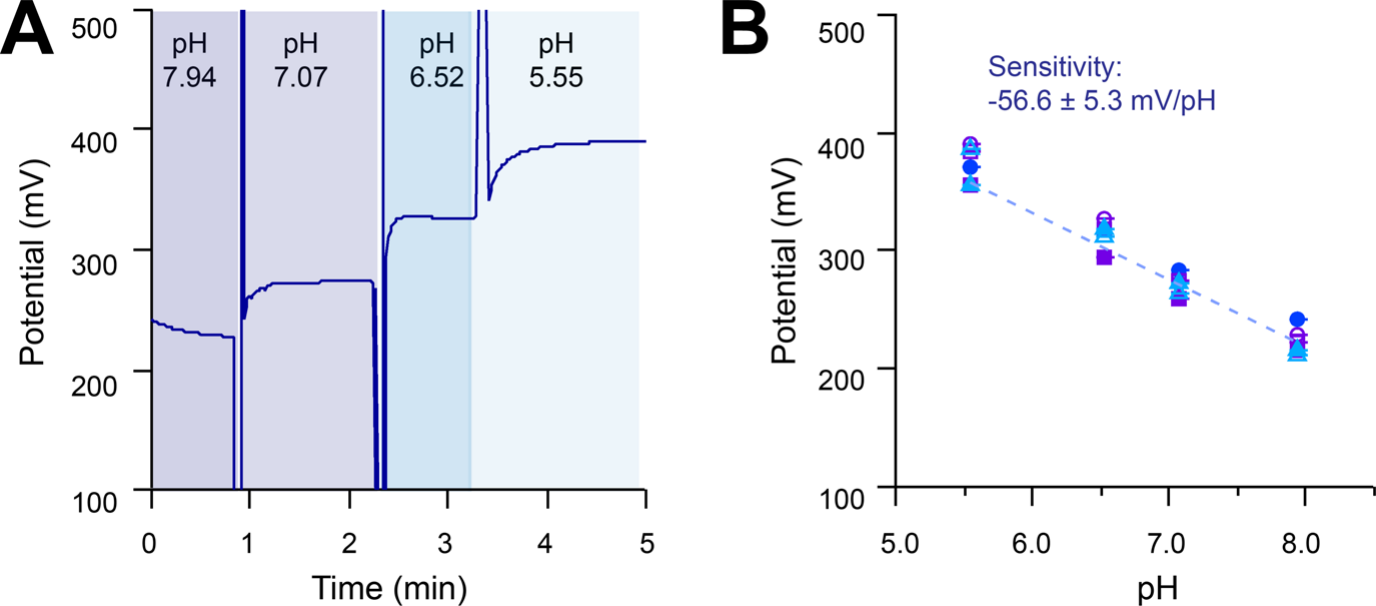
Iridium oxide-coated carbon pH sensor response to solutions with pH varying between 5 and 8. (A) Open circuit potential vs time trace showing a typical signal, where potential is measured vs an Ag|AgCl reference electrode. (B) pH vs open circuit potential for fabricated sensors. Three different colored markers represent three different pH sensors (three different electrodes) within the same fiber, and open vs filled markers indicate repeats of the same sensor. Averaged data are fitted using a linear model (dashed line). A consistent change in potential is observed as the solution is changed.

For pH sensing, a carbon electrode was coated with iridium oxide. The application of iridium oxide-based pH sensors has been studied for over three decades and shows great potential.^32,33^ We tested the pH response in the physiological range using PBS solutions of varying pH, Figure 3A. A linear relationship is observed between the open circuit potential of the iridium oxide-coated electrode and pH value, within the pH range 5 and 8. The slope of this line is close to Nernstian behavior, and response is repeatable, Figure 3B, with similar sensitivities to literature sensors.^33^ The pH sensors remained stable during experiments with continuous use (1 h). Some variability is seen between sensors, Figure 3B, and between sensors on different fibers, Figure S4A; however, repeats with the same sensor show excellent reproducibility. This demonstrates that the fiber platform can be used to fabricate pH sensors.

### Fibers as Lactate Sensors

**Figure 4:**
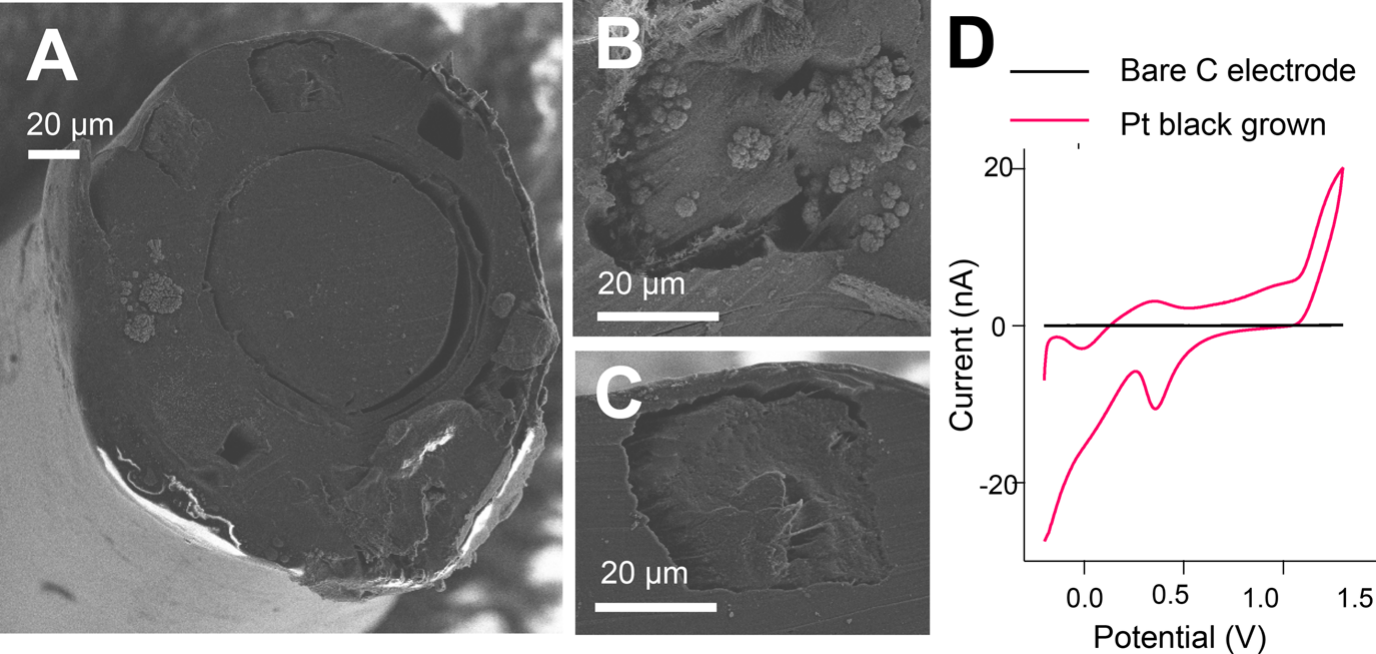
SEM images showing a (A) multifunctional fiber surface after platinum black deposition on one electrode, (B) zoomed-in section of a fiber with an electrode with platinum black grown, and (C) zoomed-in section of a fiber with a bare electrode where no potential was applied while in Pt black growth solution. (D) CVs of two electrodes from a fiber in sulfuric acid (0.5 M H_2_SO_4_, 100 mV s^–1^ vs Ag|AgCl). A flat response was obtained from the bare carbon electrode (black line), whereas the characteristic CV peaks of Pt are seen following platinum black growth (red line).

The amperometric lactate sensor platform uses lactate oxidase to oxidize lactate, producing hydrogen peroxide in the process. Hydrogen peroxide is then detected amperometrically on the electrode surface. As platinum is a better electrocatalyst for peroxide oxidation than carbon, potentiometry was used to grow platinum black on the carbon surface. Despite the whole electrode immersion in solution, platinum black is only grown on the electrochemically activated surface. This resulted in regional growth rather than a continuous film, Figure 4A,B. The resulting cyclic voltammograms (CVs) show platinum-like oxidation and reduction peaks and an increase in surface area, Figure 4D.

**Figure 5:**
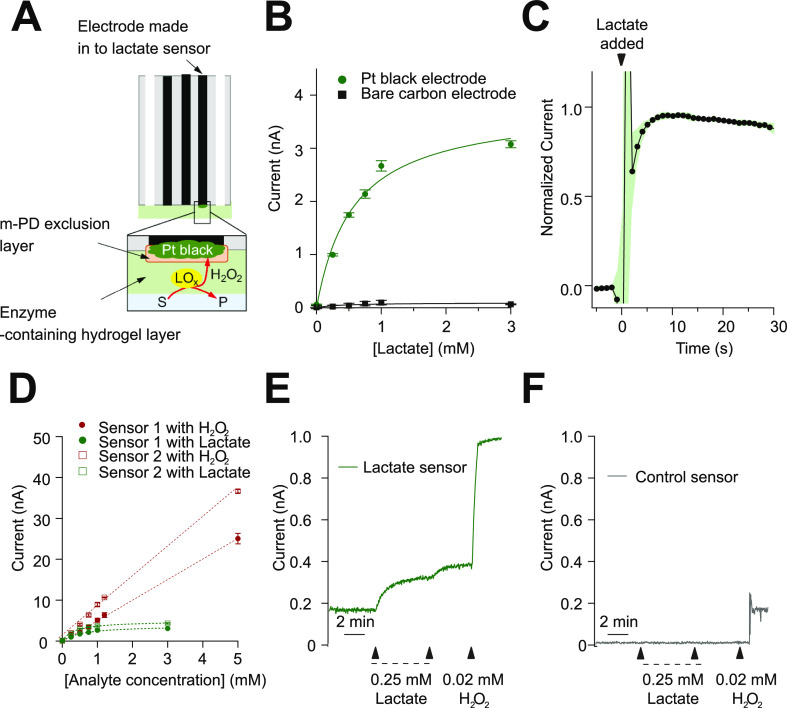
(A) Schematic of the lactate electrode biosensor on a fiber. (B) Response to lactate of a lactate sensor made from platinum black-coated electrodes and a nonfunctionalized carbon electrode. Data are fitted with the Michaelis–Menten equation. Markers represent mean ± standard deviation of repeated measurements (*n* = 3). (C) Normalized current response for a 0.25 mM lactate step change vs time on a Pt black-coated electrode in a stirred beaker. In black is the average response for two sensors (*n* = 12 step changes), and green represents the standard error. (D) Response of two lactate sensors to two different analytes: hydrogen peroxide (in maroon from concentrations 0–5 mM) and lactate (in green from concentrations 0–3 mM). Sensors 1 and 2 are located on the same fiber. Data are fitted with the Michaelis–Menten equation for response to lactate and a linear fit for hydrogen peroxide response. Markers represent mean ± standard deviation of repeated measurements (*n* = 3–4). (E) Current over time for a lactate sensor after the addition of lactate (two aliquots giving concentrations of 0.25 mM and then 0.5 mM lactate) and then 0.02 mM H_2_O_2_. Lactate sensors respond to both lactate and hydrogen peroxide. (F) Current recorded over time for a control biosensor (no enzyme present) after the addition of lactate (two aliquots giving concentrations of 0.25 mM and then 0.5 mM lactate), followed by 0.02 mM hydrogen peroxide. No sensor response is seen with lactate addition, response is seen with hydrogen peroxide.

A schematic of the lactate sensor is shown in Figure 5A. After platinum growth, an exclusion layer of *m*-phenylene diamine is grown via amperometry in order to prevent nonspecific signal. Successful deposition of the exclusion layer was confirmed by observing a reduced current and lack of redox peaks with a ferrocene reporter by CV, Figure S2D. The exclusion layer is followed by dip deposition of a hydrogel layer containing lactate oxidase and curing. The sensor shows greatly improved lactate detection after platinum black growth, Figure 5B, with a sensitivity change from 0.09 ± 0.01 nA/mM to 2.63 ± 0.66 nA/mM (*n* = 4) for bare carbon and Pt black-coated, respectively. This improved sensitivity is comparable to similar lactate sensors reported in the literature.^13,14,34^ The limit of detection (LOD) for our lactate sensor on a Pt black-coated electrode (calculated as the blank signal plus three times the blank signal) is 19 ± 7 μM (*n* = 4). Both the sensitivity and LOD show that the sensor is capable of measuring lactate concentrations in physiologically relevant ranges.^6,14,35^

A small amount of variability is seen in sensor response both within the same fiber and between electrodes on different fibers, Figure S4B, likely due to differences in the underlying electrode surface area and composition. Each sensor response, however, is highly reproducible. Lactate sensors remained stable over multiple measurements, with small decreases in sensitivity over time due to the enzyme component, Figure S5 and as seen in the literature.^6^ Sensor response to lactate concentration changes is on the order of a few seconds, indicating that these sensors are capable of measuring rapid transient concentration changes, Figure 5C.

When acting as a lactate sensor, the electrode is measuring hydrogen peroxide concentration as generated by lactate oxidase. Hence, it is no surprise that lactate sensors are responsive to both lactate and hydrogen peroxide, Figure 5D,E. As can be seen in Figure 5D, response is linear for hydrogen peroxide under conditions tested (up to 5 mM), while response to lactate reaches a plateau due to the lactate oxidase enzyme kinetics. Sensitivity to hydrogen peroxide is 6.14 ± 0.18 nA/mM (*n* = 2) and is higher than that to lactate (2.63 ± 0.66 nA/mM) due to the enzyme processes involved for lactate sensing. For measurement, the sensor is held at +0.7 V, at which other interferents may be oxidized. Selectivity of similar sensors with the same barrier layer has been published elsewhere and shown negligible response to serotonin, dopamine, or ascorbic acid.^13,28,35,36^ To confirm lactate is being measured without contribution from interferents, however, we fabricated control sensors. Control sensors (lacking the enzyme component required to detect lactate) show only a response to hydrogen peroxide and no response to lactate, Figure 5F.

### Fiber with a pH Sensor and Lactate Sensor for Simultaneous Detection

Electrodes are individually addressable and, hence, can be functionalized as different sensors within the same fiber. To demonstrate this, we functionalized one electrode as a potentiometric pH sensor, another as an amperometric lactate sensor, and a third with no functionalization to act as a control (bare carbon electrode). Iridium oxide was grown first, with the Pt black deposition and lactate sensor layers subsequently added. In preliminary tests, we exposed the fiber to varying pH (pH 6.52 and pH 7.56) and lactate concentration (0.1 and 0.5 mM lactate) solutions (Figure S4). Each sensor showed the expected trends, demonstrating that multiplexing with the fiber can be performed. This is observed by increases in current with lactate, decreases in potential with pH, and no changes for the control sensor. However, sensitivities are not in keeping with lactate or pH sensor calibrations on separate fibers, indicating that the electronics and methods for fabricating the sensors on the same fiber require optimization. Areas to consider and optimize include (1) the electronics for simultaneous potentiometric and amperometric measurement, (2) the processes and sequence of growth of the layers for each sensor and whether they affect one another, and (3) the mechanisms of the sensors in tandem, to be reported elsewhere.

### Applying Fiber Biosensors for In Vivo Measurements

We have demonstrated the potential of our fibers to deliver different fluids, measure fluid changes via electrochemistry, and perform pH and lactate sensing in vitro. The ideal would be to use all capabilities of the fibers for in vivo testing; however, first two areas need to be addressed: (1) improvement in fluidics as the small channel size can lead to blockages and (2) optimization of multiple sensor fabrication and measurement within the same fiber. To demonstrate application of the fibers in vivo, therefore, we chose to use the amperometric lactate sensor alone. Monitoring transient changes in lactate concentration can provide vital information about the metabolic health of the brain. For example, trauma to the brain can give rise to secondary insults such as SDs, which in turn lead to worse patient outcomes.^37^ SDs have been shown to be detrimental to the injured human brain and are accompanied by a change in concentration of important neurometabolic chemicals such as glucose, potassium, and lactate.^6,38^

**Figure 6:**
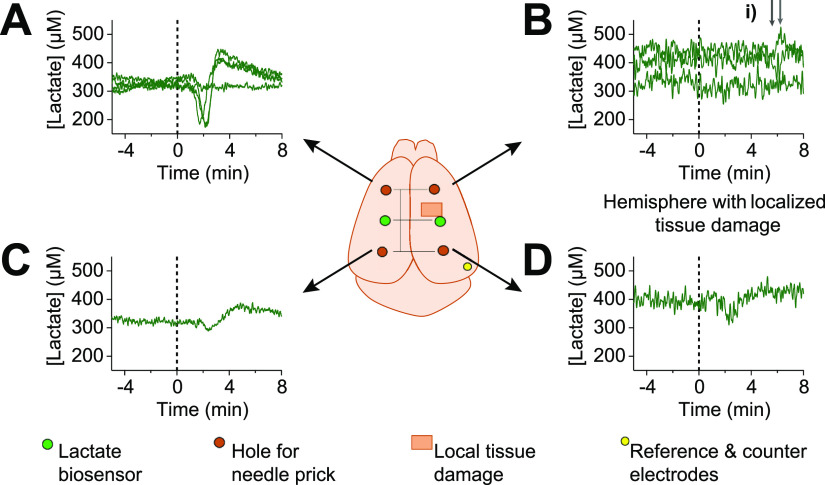
Continuous monitoring of lactate concentration in vivo in a mouse model showing the response of the lactate fiber biosensors to needle pricks from different locations. (A) Response of a lactate biosensor after needle pricks (*n* = 4) in the hole indicated from the arrow. A similar SD signature of response and delay of response is observed after each needle prick bar one (flat response); this is thought to have been an artifact of a missed needle prick, and a repeat after 10 min yielded the expected resulting signal, Figure S7. (B) No SD signature is observed after needle pricks in this location in the time frame typically seen with the other data (∼1.5 min), likely due to the local tissue damage. (i) Perhaps, a delayed response is seen in two pricks after the 6 min mark as highlighted by the small gray arrows. (C) Broadly similar pattern is seen after a needle prick in this location (*n* = 1), although slightly smaller in magnitude. (D) Again, a similar SD pattern is observed following a needle prick in this location, despite the presence of local damage near the biosensor. This indicates that tissue is still able to respond to SDs. All recordings were calibrated and time aligned to the time when the needle prick was made, represented by the black dotted line.

Here, the experiments involved needle pricks in the frontal cortex of mice to induce SDs. Of interest was whether the fiber biosensors were sufficiently sensitive to detect the propagation of the metabolic effects of SDs in a mouse brain, in particular, whether an area with local tissue damage affected the observed responses. An overview of the measurements taken for one experiment is shown in Figure 6, which shows a bird’s eye view of the mouse brain corona. A full trace for both animals of the experiments can be found in Supporting Information, Figures S7 and S8. Lactate sensor stability is predominantly influenced by loss of enzyme activity. We expect the sensors to remain usable during the experiment time period (maximum 5 h), confirmed by performing calibrations before and after implantation.

In vivo data were collected, showing that monitoring lactate concentration fluctuations was possible with the developed implanted fibers. What we believe to be the signature of an SD is observed following the majority of needle pricks. Meanwhile, in a hemisphere where a local trauma event had occurred, either a smaller or no such pattern is observed. It has been shown that ascorbate concentration fluctuates with SDs.^39^ Ascorbate is also electroactive, and at 0.7 V, it would contribute to an electrochemical signal on a bare electrode. We do not expect a signal on our barrier-coated electrode.^28^ We performed an in vivo experiment with a control sensor (lacking the enzyme component required to detect lactate) which showed no response during measurements, Figures S8A and S9. Following the experiment, the control sensor showed response to hydrogen peroxide and no response to lactate (Figure S10), as was observed in in vitro measurements with control sensors. In vivo results with the control sensor indicate that the observed response of the lactate sensors is indeed due to changes in neural lactate rather than the presence of other electroactive species such as ascorbate. In addition, this confirms that the exclusion layer of the sensor is successful in reducing interferents.

Focusing on experiment 1, the data show a time delay of 67 ± 7 s (*n* = 4 transients) between the needle prick and the change in lactate concentration, indicating that the speed of the SD wave is ∼2.7 mm/min, comparable to other reports of SD wave speeds.^8,40^ The profile for the change in lactate concentration following the needle prick is biphasic (a decrease in lactate followed by an increase). The initial decrease in concentration is likely because of the immediate energy demand following an SD, where neurons preferentially utilize lactate fuel^41^ in the presence of oxygen, to recover function. This has previously been found when the overall extracellular lactate levels are high.^42^ The subsequent increase in local lactate concentration could indicate ischemic conditions leading to anaerobic metabolism, or in response to glutamate uptake, the neurons release lactate. The observed increase in lactate concentration is in keeping with the current understanding of changes in the brain during SDs measured via rsMD^6^ and electrochemical biosensors.^13,34^ Further discussion on lactate use or relevance in the brain is beyond the scope of this work, as instead we highlight the value of real-time monitoring of lactate concentration changes.

Within a short time frame (<2 min), no change in lactate is observed when a local trauma zone is positioned between the needle prick point and the biosensor, suggesting that the physical damage affects either the movement of SDs or that the repolarization capability of the local tissue is impaired. The difference in response at different tissue locations proves that the effects measured are local effects and not general global changes in lactate concentrations. It may indicate that the path of SDs can change due to the presence of localized damage, as discussed by Nakamura, Graf, et al.^43^ However, because only two experiments were performed and there is variation within the biosensor and animal response, this cannot be statistically verified. We note that these flexible fibers with lactate biosensors successfully monitor local concentration changes in in vivo lactate, demonstrating, for the first time, their potential as directly implantable biosensors.

## Conclusions

We applied flexible polymer fibers containing multiple graphite-doped electrodes and microfluidic channels to create electrochemical biosensors for pH and lactate. Changes in neural lactate were detectable in vivo.

Initially, the electrochemical behavior of the fiber electrodes was investigated. This was then coupled with flowing electroactive and buffer solutions through the microfluidic channels while being monitored amperometrically with fiber electrodes. The preliminary data demonstrate a potential use by coupling the fluidic component with electrochemical measurements at the electrodes; with development one could perfuse and measure a compound of interest at the fiber surface. Potentiometric sensors were demonstrated by making the electrodes responsive to pH, demonstrating a possible use of electrodes as sensors. Amperometric lactate sensors were developed and shown to respond to lactate concentration in vitro. Variability between electrodes/sensors on the same fiber and across fibers have been examined for bare electrodes (Figure S2A–C), pH sensors (Figure S4A), and lactate sensors (Figure S4B), showing adequate reproducibility. Preliminary experiments with a pH and lactate sensor within the same fiber were performed, demonstrating multiplexing capability. Lactate sensors were then translated in vivo, where they were used to monitor the change in local lactate concentration in response to SDs following brain needle pricks in a mouse model, supported by no response observed in control sensors. A number of needle pricks resulted in repeatable signatures of response which we attribute to SDs. When local tissue damage was present, the response was shown to change from what was previously observed, suggesting that local changes may be monitored using the fiber biosensors. In vivo application demonstrates the potential of flexible fibers for making implantable biosensors.

Future directions are aimed at miniaturizing the fiber and including local field potential (LFP) measurements at one of the electrode contacts.^44^ LFP measurements allow confirmation of the timing of a passing SD wave. This capability will complement lactate sensing and confirm that the observed lactate changes are from passing SDs. Investigation of in vivo pH sensing will also be performed, to be reported elsewhere. Furthermore, future directions involve experiments that encompass the multifunctionality and multiplexing capability of the fibers by making each electrode into a different sensor and by the delivery of drugs of interest, for example, dexamethasone, to reduce penetration injury during implantation.^8^ These experiments will require optimization of sensors within the same fiber and improvement in fluidics, as the small microfluidic channels are subject to blocking. Once larger and robust microfluidic channels are included, we believe solution perfusion will be possible even with the added sensor layers such as the lactate sensor hydrogel layer, based on our experiments on similar, larger devices (data not shown). The next stage is hence to optimize and then use the full capabilities of these fibers and carry out multimodal analysis.

## Supporting Information Available

Image of the fiber device, cyclic voltammograms of multiple bare electrodes and fibers before and after cutting, bare electrode and after growth of the exclusion layer, current time trace of electrodes during PBS flow through the microfluidic channel, calibrations for multiple pH and lactate sensors, lactate sensor response over time, preliminary data showing pH and lactate simultaneous sensing in a single fiber, full-time traces of the in vivo experiments, and equations used for fitting and calculating the electrode surface area (PDF)
