# Supplementary material for: Fiber-Based Electrochemical Biosensors for Monitoring pH and Transient Neurometabolic Lactate
Source: Anal Chem. 2021 Apr 2;93(17):6646–55. doi: 10.1021/acs.analchem.0c05108 (PMC8153388; doi:10.1021/acs.analchem.0c05108)
Supplement: Supplementary file 1 — ac0c05108_si_001.pdf [file ac0c05108_si_001.pdf]

Fiber-based electrochemical biosensors for monitoring pH and transient neurometabolic lactate

Marsilea A. Booth,[a,b,c] Sally A. N. Gowers,[a] Melinda Hersey,[d] Isabelle C. Samper,[a] Seongjun Park,[e,f,g] Polina Anikeeva,[h] Parastoo Hashemi,[b,d] Molly M. Stevens,[a,b,c]* Martyn G. Boutelle[a]*

[a]Department of Bioengineering, Imperial College London, UK

[b]Department of Materials, Imperial College London, UK

[c]Institute of Biomedical Engineering, Imperial College London, U.K.

[d]Department of Chemistry, University of South Carolina, SC, US

[e]Department of Electrical Engineering and Computer Science, Massachusetts Institute of Technology, US

[f]Department of Bio and Brain Engineering, Korea Advanced Institute of Science and Technology (KAIST), Republic of Korea

[g]KAIST Institute for Health Science and Technology, Republic of Korea

[h]Department of Materials Science and Engineering, Massachusetts Institute of Technology, US

Corresponding authors

*E-mails: Molly M. Stevens m.stevens@imperial.ac.uk, Martyn G. Boutelle m.boutelle@imperial.ac.uk

Table of Contents

FIGURES S-2

Figure S1. An image of a connectorized multifunctional fiber. S-2

Figure S2. Cyclic voltammograms (CVs) of bare electrodes on different fibers, and CVs before and after barrier layer growth. S-2

Figure S3. Current time traces at 2 bare electrodes while PBS solution is flown through microfluidic channel. S-3

Figure S4. Response of four pH sensors and four lactate sensors. S-3

Figure S5 Response of a lactate sensor over time and subsequent change in sensitivity. S-4

Figure S6. Preliminary results showing simultaneous lactate and pH sensing. S-5

Figure S7. Full trace for *in vivo* experiment 1 performed in a mouse model. S-6

Figure S8. Full trace for *in vivo* experiment 2 performed in a mouse model. S- 7

Figure S9. Overlayed trace after needlepricks at different time points for experiment 2. S-8

Figure S10. Response of the control biosensor used *in vivo* to lactate and hydrogen peroxide. S-8

EQUATIONS S-9

Equation S1 S-9

Equation S2 S-9

FIGURES

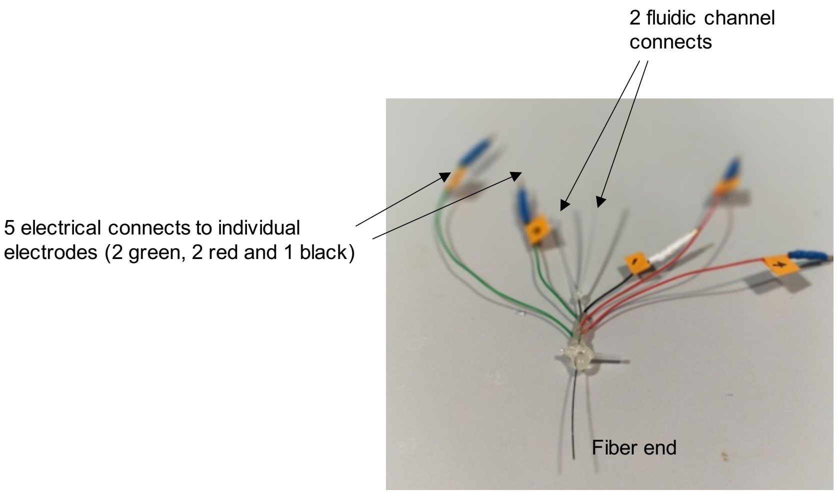

Figure S1. An image of a connectorized multifunctional fiber with 5 of the 6 electrodes electrically connected and two fluidic channels connected.

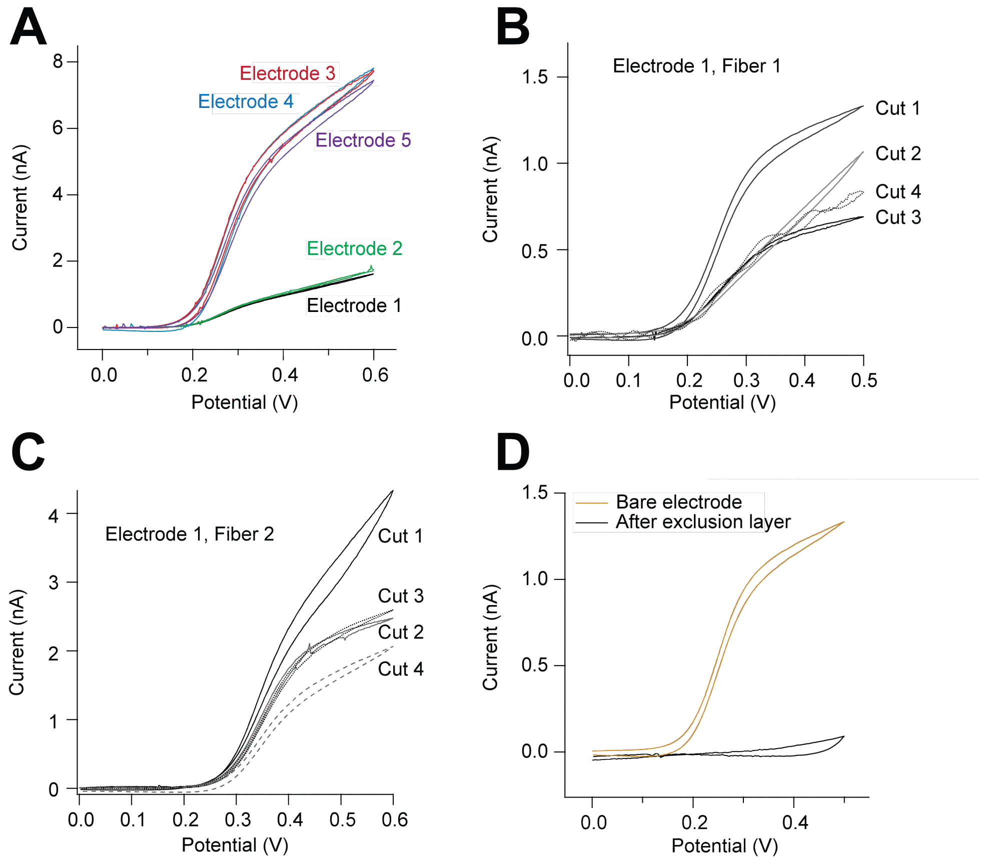

Figure S2. A) Cyclic voltammograms (CVs) of 5 bare electrodes within the same fiber. Variability is likely due to the geometric differences in electrodes along with the inhomogeneity in the composite. B) CVs after multiple cuts with a razor blade of a fiber labelled 'Fiber 1', exposing a new surface each cut. As can be seen, the electrode is variable in response through the fiber length. C) CVs of an electrode after multiple cuts with a razor blade of a second fiber labelled 'Fiber 2'. D) CVs showing response before (orange) and after (black) growth of a barrier layer of *m*-phenylene diamine on the electrode. The reduced current and lack of redox peaks indicate the barrier layer has been successfully grown to exclude the redox reporter. All CVs are in ferrocene monocarboxylate solution (1.5 mM, 10 mVs-1 vs Ag|AgCl).

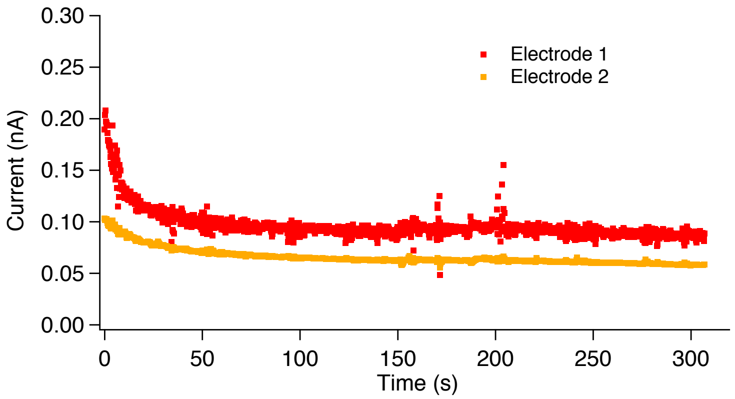

Figure S3. Current time traces measured at two electrodes in a single fiber containing two microfluidic channels, as described in manuscript Figure 2. At time 0 channel 2 containing Fc solution is stopped and channel 1 flows PBS solution resulting in a corresponding decrease followed by a stable flat current as faradaic processes are no longer a major contributor to current response.

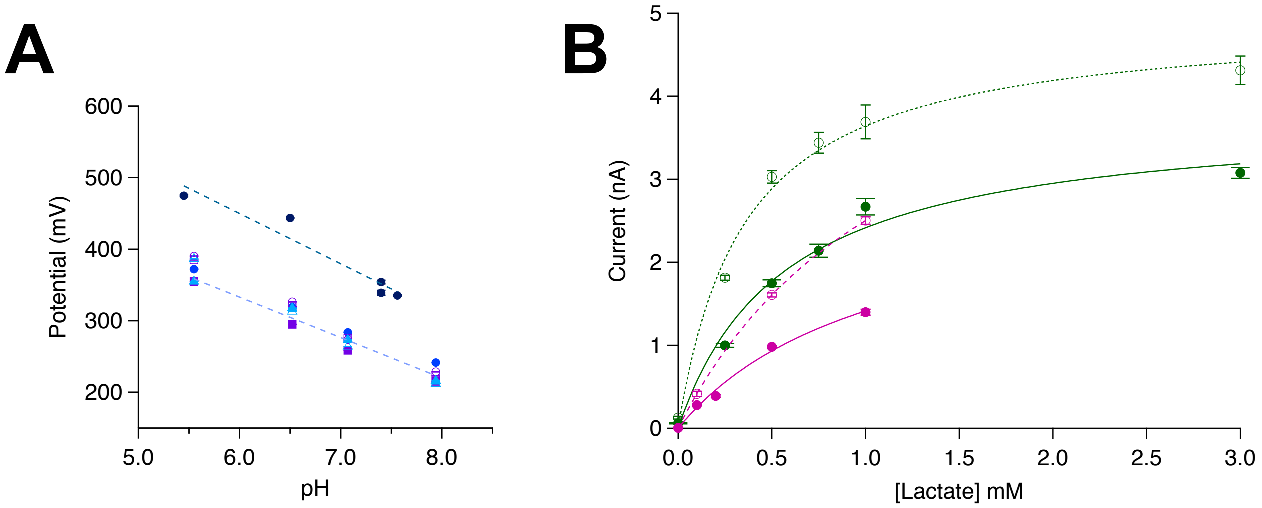

Figure S4A) Response to pH of four individual pH sensors across two fibres (with one fibre represented by dark blue and the other light blues). The single pH sensor represented by dark circles has a lactate sensor on the same fibre, hence the difference in potential values is likely due to a the added fibre layers/differences to ground/reference. Data is fitted with a linear fit. Although the absolute potentials vary, the sensors display similar sensitivities (-56.6 ± 5.3 vs -71.1 ± 14.4 mV/pH). B) Response to lactate of four individual lactate sensors made from platinum black coated electrodes. Results from two fibres are compared (with each fibre represented by a different colour, one green and one pink). Two sensors per fibre device are compared with one sensor represented by open circles, and the other filled. Data is fitted with the Michaelis–Menten equation. Markers represent mean ± standard deviation of repeated measurements (n = 2-3).

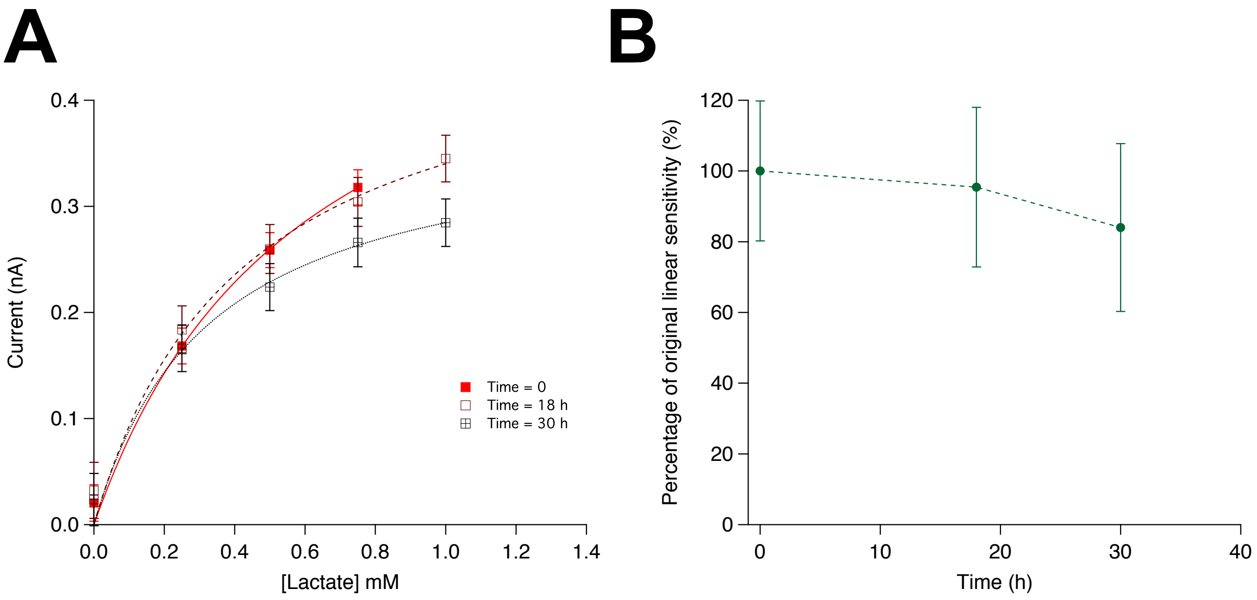

Figure S5 A) Response to lactate of one single fibre lactate sensor with calibrations at different time points. The first calibration is considered time point 0 shown by red squares, after 18 h another calibration is shown by red open squares and after 30 h the calibration is shown by black open markers. Between these time points the sensor was used for 2 h as a sensor and subsequently stored in the freezer. A decrease in sensor performance is observed over time, likely due to natural degradation of the enzyme in the sensor. B) The decrease in sensitivity (calculated from the first 3 points of each data set in A) shows a decrease in sensitivity over time and with use. Error is derived from the error in sensitivity measurements, and the line is drawn to guide the eye.

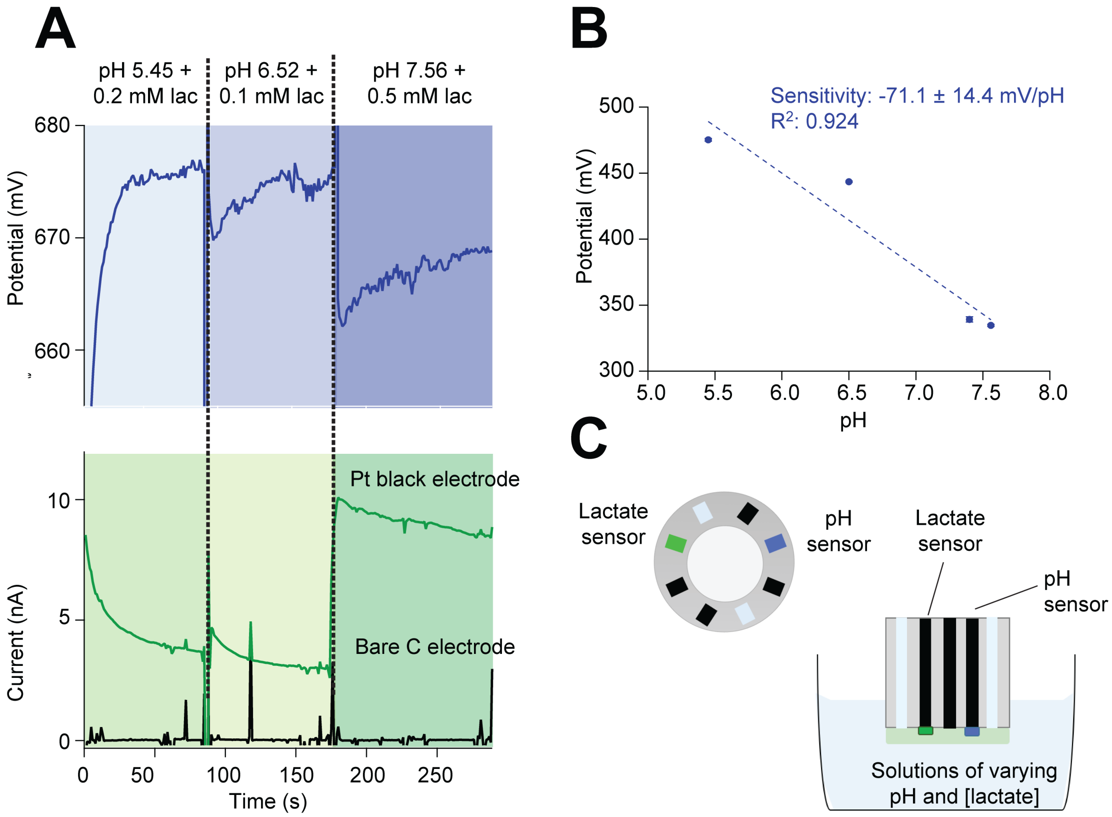

Figure S6. A) Potential and current responses to three solutions possessing different lactate concentrations and pH (labelled above) as measured at the pH sensor (blue trace), lactate sensor (labelled Pt black electrode, green trace) and control sensor (labelled bare carbon electrode, black trace). Expected trends are seen for all three electrodes demonstrating multiplexing with our fiber is possible. B) Calibration curve for the pH sensor measured individually. As can be seen when comparing A and B, a large jump in potential occurs when both amperometric and potentiometric sensors are connected, as well as a change in sensitivity. Additionally, a linear fit may not best describe this preliminary proof-of-concept data. Both the electronics and methods for fabricating the pH and lactate sensor on the same fiber require optimization. C) Schematic showing end on and side view of the set-up to test a fiber with a pH and lactate sensor for simultaneous measurement of pH and lactate.

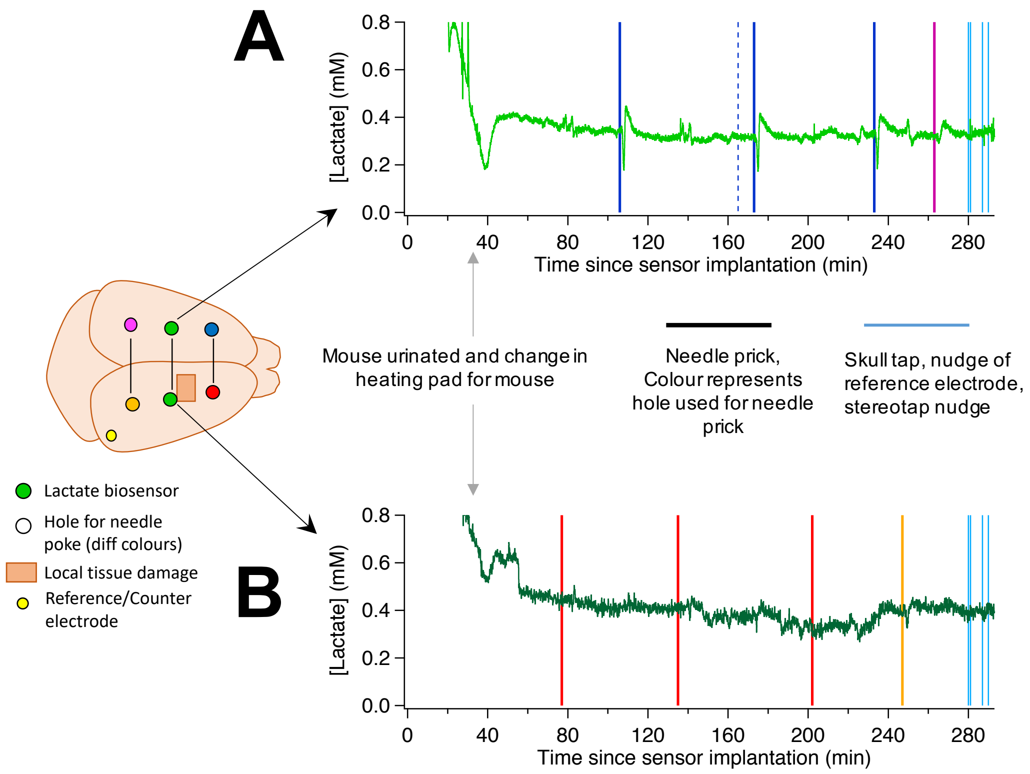

Figure S7. Full traces of experiment 1, *in vivo* experiment performed in a mouse model. A) Lactate concentration vs time trace for the lactate biosensor in the hemisphere where no prior damage was present. Needle pricks are represented by a line and the colour represents which hole was used for the needle prick. No SD wave was observed following the second needle prick (blue dashed line). Therefore, a repeat needle prick after 10 min was performed in case an error had occurred. The expected SD wave followed. B) Lactate vs time trace for the lactate biosensor in the hemisphere where local tissue trauma was present. Needle pricks are represented by a line and the colour represents which hole was used for the needle prick. At the end of the experiment control tests were performed, represented by light blue lines. These included a skull tap, nudge of reference electrode and stereotax nudge. No change in lactate concentration was observed.

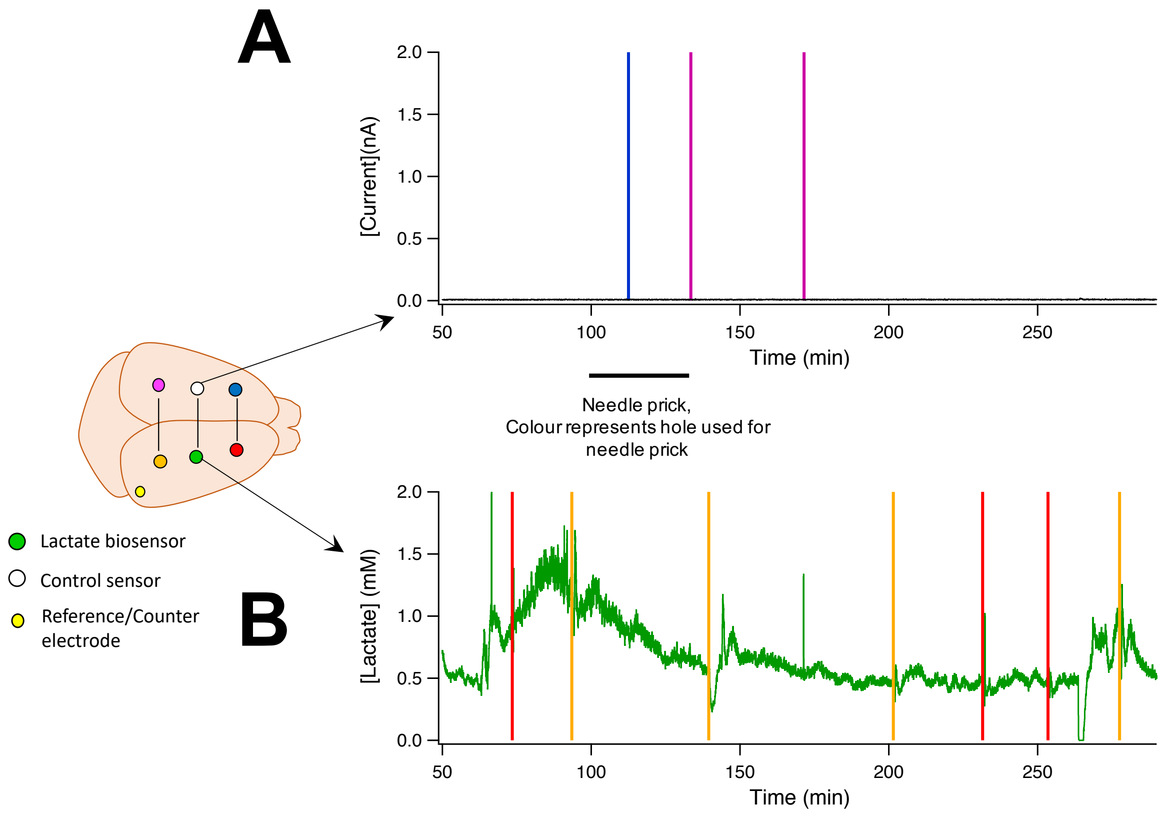

Figure S8. Full traces of experiment 2, *in vivo* experiment performed in a mouse model with one lactate sensor and one control electrode. A) Current vs time trace for the control biosensor in the hemisphere where no prior damage was present. Needle pricks are represented by a line and the colour represents which hole was used for the needle prick. The control electrode was shown *in vitro* to respond to hydrogen peroxide but not lactate. The lack in signal indicates the biosensors are responding to a change in lactate concentration and not the presence of hydrogen peroxide or other electroactive species. B) Lactate vs time trace for the lactate biosensor. Needle pricks are represented by a line and the colour represents which hole was used for the needle prick. An initial increase in lactate can be seen peaking at 85 min. This is likely due to the mouse health deteriorating; at 64 min it was noted mouse health was poor and heart palpitations were required, as well as a new heating pack at 66 min. Following this the lactate signal seemed to stabilize and hence the experiment was continued.

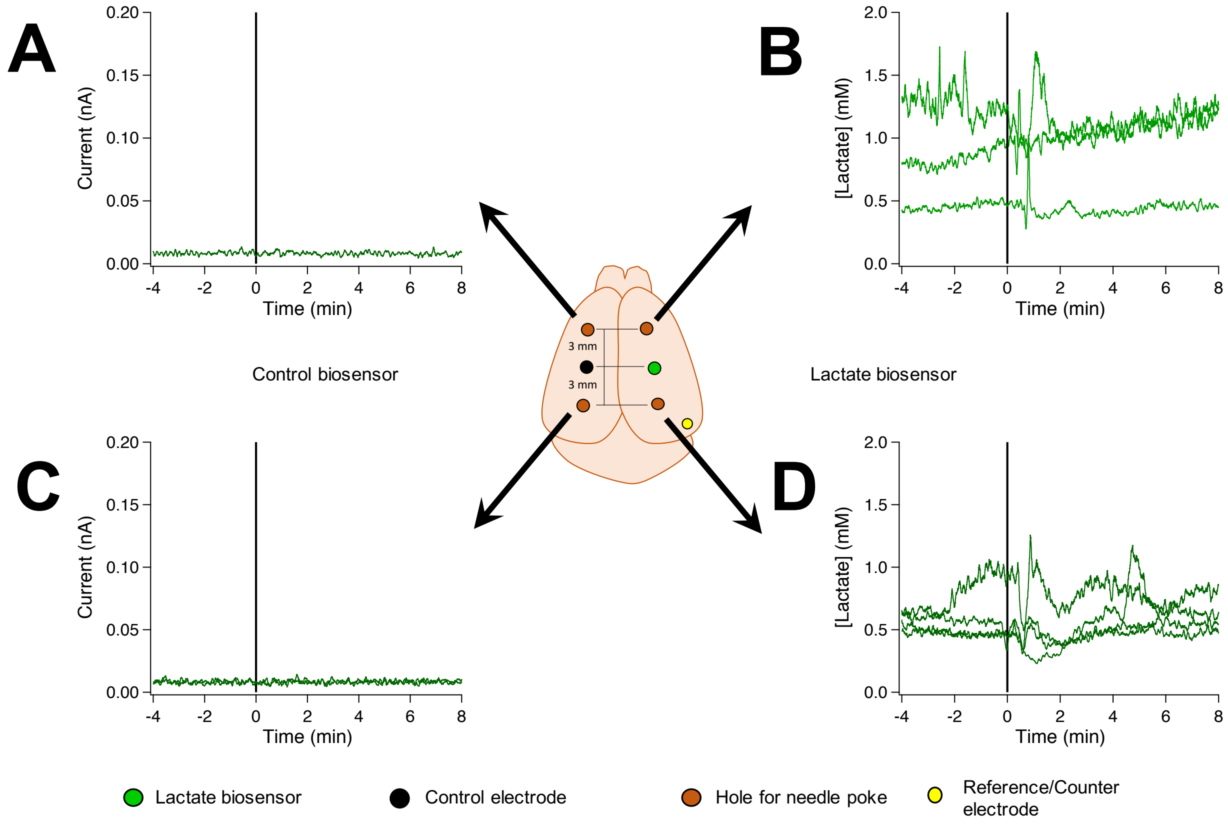

Figure S9. Continuous monitoring of lactate concentration *in vivo* comparing the response of a lactate fiber biosensor to that from a control electrode (no lactate oxidase present) following needle pricks from different locations. A) The response of a control electrode after needle pricks (n = 1) in the hole indicated. No response is observed. B) A possible SD pattern is observed in some of the response measured by the lactate biosensor following a needle prick in this location (n = 3). C) No response is seen after a needle prick in this location (n = 2) at the control electrode. D) Again, an SD pattern may be present in some of the needle pricks in this location (n = 4). All recordings were calibrated, and time aligned to the time when the needle prick was made, represented by the black line.

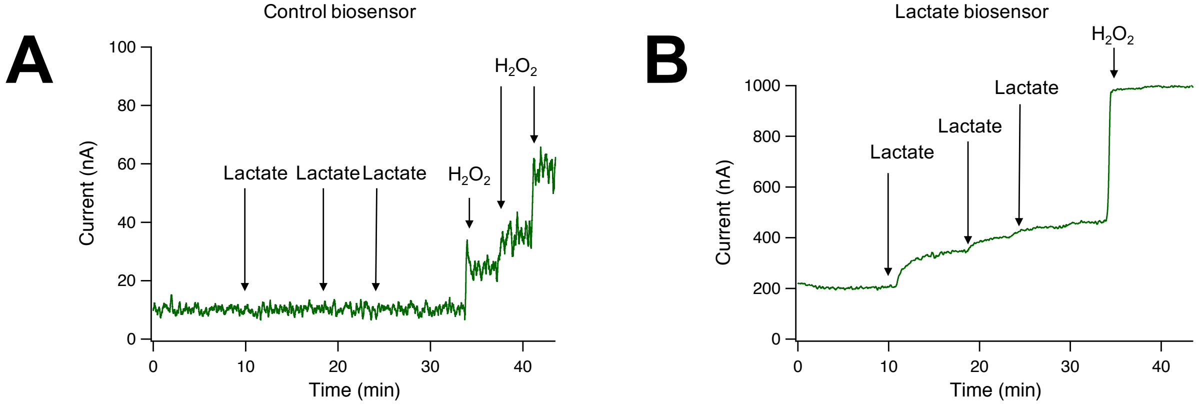

Figure S10. A) Response of the control biosensor used *in vivo* to lactate and hydrogen peroxide. No response is seen to lactate, while hydrogen peroxide gives rise to a change in current. B) Response of the lactate biosensor used *in vivo* to lactate and hydrogen peroxide. Response is seen for both lactate and hydrogen peroxide.

EQUATIONS

Equation S1

Michaelis–Menten equation, used to fit lactate biosensor response.

$$v = \frac{V_{max}[lac]}{K_M + [lac]}$$

Where v is the reaction rate (described by current), V_{max} is the maximum reaction rate achieved by the system (at saturating concentration and shown by maximum current), $[lac]$ is the concentration of lactate and K_M is the Michaelis constant.

Equation S2

Assuming a disc area of radius r (cm), steady state current would be expected to be given by:

$$I = 4nFDrc$$

Where F is the Faraday constant ($Cmol^{-1}$), D ($cm^2\ s^{-1}$) is the diffusion coefficient of the oxidized analyte, r is the radius (cm) and c is the bulk concentration of the analyte ($mol\ cm^{-3}$). Scan rate in this case is 10 mVs^{-1}.

Using observed limiting currents for two electrodes, as well as the following values for the used solution D = $5.73 \times 10^{-6}\ cm^2s^{-1}$ and c = $1.5 \times 10^{-6}\ mol\ cm^{-3}$, electrode radii are estimated as:

Electrode 1 I = 1.21 ± 0.1 nA

r = 3.65 μm

Electrode 2 I = 1.73 ± 0.11 nA

r = 5.21 μm

This corresponds to areas of 41.9 and 85.3 $μm^2$ for electrodes 1 and 2, respectively.
